# Statistical analysis plan for the Petal trial: the effects of parental touch on relieving acute procedural pain in neonates

**DOI:** 10.12688/wellcomeopenres.19819.3

**Published:** 2024-12-18

**Authors:** Luke Baxter, Annalisa G.V. Hauck, Aomesh Bhatt, Maria M. Cobo, Caroline Hartley, Simon Marchant, Ravi Poorun, Marianne van der Vaart, Rebeccah Slater

**Affiliations:** 1 Department of Paediatrics, University of Oxford, Oxford, England, UK; 2 Colegio de Ciencias Biologicas y Ambientales, Universidad San Francisco de Quito USFQ, Quito, Ecuador; 3 University of Exeter Medical School, University of Exeter, Exeter, England, UK; 4 Department of Paediatrics, Royal Devon University Healthcare NHS Foundation Trust, Exeter, England, UK

**Keywords:** nociception, RCT, analgesia, stroking, PIPP-R, biomarker

## Abstract

**Background:**

Infants undergo multiple clinically-required painful procedures during their time in hospital, and there is an increasing desire from both parents and clinical staff to have parents directly involved in their newborn’s pain relief. To avoid biases due to selective analysis and reporting, a clinical trial’s statistical analysis plan (SAP) should be finalised and registered prior to dataset lock and unblinding. Here, we outline the SAP for the Petal trial, which was registered on the ISRCTN registry prior to dataset lock and unblinding.

**Methods:**

The Petal trial is a multicentre, individually randomised, parallel-group interventional superiority trial. The study involves in-patient neonates born at or after 35+0 weeks gestation with a postnatal age of ≤7 days, in two hospital research sites (John Radcliffe Hospital, Oxford, UK; Royal Devon and Exeter Hospital, Exeter, UK). The primary objective is to investigate the potential efficacy of a non-pharmacological parent-led stroking intervention on reducing the magnitude of neonates’ noxious stimulus-evoked brain activity. The primary outcome is the neonate’s brain activity recorded using electroencephalography (EEG) in response to a heel lance blood sampling procedure. Secondary outcomes include neonatal clinical pain scores and tachycardia, and parental anxiety. The study hypothesis is neonates’ pain responses and parents’ anxiety scores are lower in the intervention group. Randomisation will be via a minimisation algorithm to maintain balance in five prognostic factors.

**Conclusions:**

Paediatric pain trials have been highlighted by regulatory bodies as an important and challenging topic, with interest increasing in brain imaging outcomes. The Petal trial, to which this SAP relates, is part of a larger effort of establishing a brain-based EEG outcome measure of infant pain for use in clinical trials. This SAP is thus likely to be of interest to those in academia, pharmaceutical companies, and regulatory bodies.

**Trial registration:**

ClinicalTrials.gov: NCT04901611, 25/05/2021; ISRCTN: ISRCTN14135962, 23/08/2021).

## Introduction

### Background and rationale

A statistical analysis plan (SAP) provides a detailed outline of the statistical principles and analyses that are to be employed when analysing a clinical trial dataset. The SAP should include sufficient information to support replication by independent researchers ( Baker, 2016), and should be finalised and made publicly available prior to data unblinding to avoid biases that can be introduced due to selective analysis and reporting ( Dwan *et al*., 2014; Kirkham *et al*., 2010). To support these goals and help establish best practice in SAP development and reporting, JAMA published in 2017 a guidance document and checklist for statistical analysis plans in clinical trials ( Gamble *et al*., 2017).

The present SAP is a technical extension of the Petal trial protocol, which is published elsewhere ( Cobo *et al*., 2022). Our SAP is structured according to the JAMA guidelines ( Gamble *et al*., 2017), and follows the principles of the International Council for Harmonisation (ICH) guidelines E3, E6, and E9 ( ICH, 2023). Our SAP was finalised on 30 March 2023 and uploaded to the ISRCTN registry on 31 March 2023 ( https://doi.org/10.1186/ISRCTN14135962), hard locking of the database occurred on 04 April 2023, and data unblinding occurred subsequently on the same day.

This SAP outlines the analysis plan for the Petal trial, which investigates the effects of parental touch during an acute skin-breaking clinical procedure on infant pain indicators and parental anxiety. The clinically required skin breaking procedure is a heel lance for blood sampling. The parental touch intervention is stroking of the ipsilateral leg prior to the heel lance. This type of stroking is an instinctive parental behaviour, which may provide a simple parent-led intervention to alleviate acute procedural pain. This intervention may reduce noxious input reaching the brain, potentially through activating neonates' C-tactile (CT) fibre system ( Gursul *et al*., 2018; Liljencrantz *et al*., 2017; von Mohr *et al*., 2018).

### Objectives

The objectives of the Petal clinical trial are outlined in Table 1. The primary outcome is the magnitude of the heel lance-evoked brain activity. The brain activity is recorded using electroencephalography (EEG), and the outcome is quantified as the magnitude of a noxious-specific waveform scaled to the evoked response ( Hartley *et al*., 2017). The waveform is a neurodynamic response function, analogous to the haemodynamic response function (HRF) of fMRI; throughout this SAP, we refer to the noxious neurodynamic response function as n-NRF. Due to the subjective nature of pain and the non-verbal nature of neonates, there is no gold standard pain measure for this population. While our primary outcome (the n-NRF magnitude) neither directly reflects nociception, the neural process of encoding noxious stimuli ( IASP, 2024; Raja *et al*., 2020), nor pain experience, the unpleasant sensory and emotional subjective experience ( IASP, 2024; Raja *et al*., 2020), it is a pertinent and accessible feature of central importance to understanding neonates’ neural responses to noxious input and the neurophysiology of the early developing pain system. Additionally, the n-NRF magnitude is a validated and reproducible pain-relevant measure ( Aspbury *et al*., 2024; Hartley *et al*., 2017) that has demonstrated responsiveness to analgesic intervention ( Cobo *et al*., 2021). With these considerations in mind, for consistency with established use of terminology, we refer to the n-NRF magnitude as a pain outcome throughout. This is consistent with pain terminology used in the field of neonatal pain assessment, for brain-based measures in both observational studies ( Olsson *et al*., 2016; Rupawala *et al*., 2023) and clinical trials ( Cornelissen, 2016; Wang, 2020).

There are three secondary outcomes. The two neonatal secondary outcomes are the Premature Infant Pain Profile Revised (PIPP-R) scores and tachycardia; the one parental secondary outcome is post-procedural anxiety. PIPP-R is a commonly used validated clinical pain tool that incorporates measures of heart rate, oxygen saturation, and facial expression change ( Stevens *et al*., 2014). The tachycardia outcome is binary, indicating whether the neonate became tachycardic in response to the procedure or not. The parental post-procedural anxiety is assessed using the State-Trait Anxiety Inventory-State (STAI-S) score ( Spielberger *et al*., 1983). For all primary and secondary outcomes, the null ( *H*_0_) and alternative ( *H*_1_) hypotheses are as follows. *H*_0_ : μ _1_ = μ _2_ *or OR*_1_ = *OR*_2_; *H*_1_ : μ _1_ ≠ μ _2_ *or OR*_1_ ≠ *OR*_2_, where μ _1_ and μ _2_ are the means (for EEG, PIPP-R, and STAI-S) and *OR*_1_ and *OR*_2_ are the odds ratios (for tachycardia) of the intervention and control groups.

**Table 1.** Objectives and outcome measures. Abbreviations: EEG = electroencephalography; PIPP-R = Premature Infant Pain Profile – Revised; STAI-S = State-Trait Anxiety Inventory – State.

	Objectives	Outcome measures
**Primary**	To test whether parental stroking prior to a clinically required heel lance reduces noxious stimulus-evoked brain activity assessed using EEG following the heel lance	Magnitude of the noxious stimulus-evoked brain activity following the heel lance (EEG data recorded in the 1000 ms period following each heel lance).
**Secondary**	To test whether parental stroking prior to a heel lance reduces clinical pain scores (PIPP-R) following the heel lance	PIPP-R score during the 30 s period post-heel lance.
	To test whether parental stroking prior to a heel lance reduces incidence of becoming tachycardic following a heel lance	Tachycardia in the 30 s period post-heel lance.
	To test whether parental stroking prior to a heel lance reduces parental anxiety, compared with post-procedural stroking.	STAI-S scores post procedure.

The following Methods section is structured to reflect the JAMA SAP guidelines, and thus includes four subsections: study methods, statistical principles, trial population, and analysis. This research note outlines the key points in each subsection. Further details on each topic can be found in our SAP document on the ISRCTN registry ( https://doi.org/10.1186/ISRCTN14135962). The closing Discussion section of this research note highlights the value and importance of the current SAP to the broader research community, including those in academia, pharmaceutical companies, and regulatory bodies.

## Methods

### Study methods

***Trial design and framework*.** The Petal trial is a multicentre, individually randomised, parallel-group interventional trial, with two research sites (John Radcliffe Hospital, Oxford, UK, and Royal Devon and Exeter Hospital, Exeter, UK). Participants will be studied on a single test occasion while they are in hospital. Participants will be included in the study for approximately one hour: 30 min before and after the time when the heel lance is performed.

This is a superiority trial comparing standard of care and parental stroking performed before a heel lance (the intervention group) to standard of care alone before a heel lance (the control group). Note, in the control group, the parents also stroked their baby after the heel lance and after the baby’s responses to the heel lance were recorded; thus, the post-procedural parental stroking in the control group had no impact on the heel lance procedure or the recorded responses to the heel lance. The heel lance procedure involves using a small, spring-loaded lancet with a blade that, within milliseconds, creates a small cut that allows blood to be collected for testing. The parental stroking intervention is performed at approximately 3 cm/s for 10 seconds down the lower limb receiving the heel lance. During the intervention, the parent will follow an animation which shows the correct speed of stroking. The same animation will be used to train parents and to guide their stroking in both trial arms. In both sites, standard of care involves offering all neonates comfort measures including swaddling and non-nutritive sucking, in line with local practice guidelines.

***Randomisation*.** Randomisation of participants to the intervention arm or control arm will be managed via a secure web-based randomisation facility provided by Sealed Envelope Ltd, UK. Participants will have a roughly equal chance of being allocated to either arm (allocation ratio of 1). The randomisation program will use a minimisation algorithm to ensure approximate balance between the groups with respect to five minimisation variables that are likely to cause meaningful variability in pain response estimates: gestational age at birth, postnatal age at time of randomisation, sex, the primary indication for blood sampling, and research site. The minimisation approach was chosen due to its capacity to balance multiple prognostic variables in trials with small sample sizes, which would unlikely achieve balance through pure randomisation approaches.

***Sample size*.** Sample size calculations are based on data from previous studies investigating the effect of (experimenter-led) soft brushing of the skin at CT-optimal rate on the response to a noxious stimulus or clinical heel lance in term neonates ( Cobo *et al*., 2021; Gursul *et al*., 2018). From these studies, the mean brain activity magnitude evoked by heel lancing in the control group is estimated to be 1.07 with a standard deviation (SD) of 0.66. In neonates who received soft brushing of the skin before the heel lance procedure, Gursul and colleagues ( Gursul *et al*., 2018) observed a 40% reduction, and Cobo and colleagues ( Cobo *et al*., 2021) observed a 39% reduction, in heel lance-evoked brain activity. In adult studies, similar reductions in noxious-evoked brain activity are accompanied by significantly lower verbal pain scores ( Lorenz *et al*., 1997; von Mohr *et al*., 2018). Reductions of 30%-36% in pain intensity ratings are considered clinically "meaningful" as reported by a consensus statement for adult clinical pain trials ( Dworkin *et al*., 2008). Therefore, we consider a 40% reduction in the intervention group to be a clinically significant outcome in this trial. Thus, the intervention arm heel lance-evoked mean brain activity magnitude is set at 0.642 and SD is 0.66. Assuming a t-distribution, with 90% power, a two-sided 5% significance level, and an allocation ratio of 1, we estimated a sample size of n=102 infants. Allowing for approx. 10% loss due to technical difficulties or clinical issues, the final sample size is n=112.

**Figure 1.**
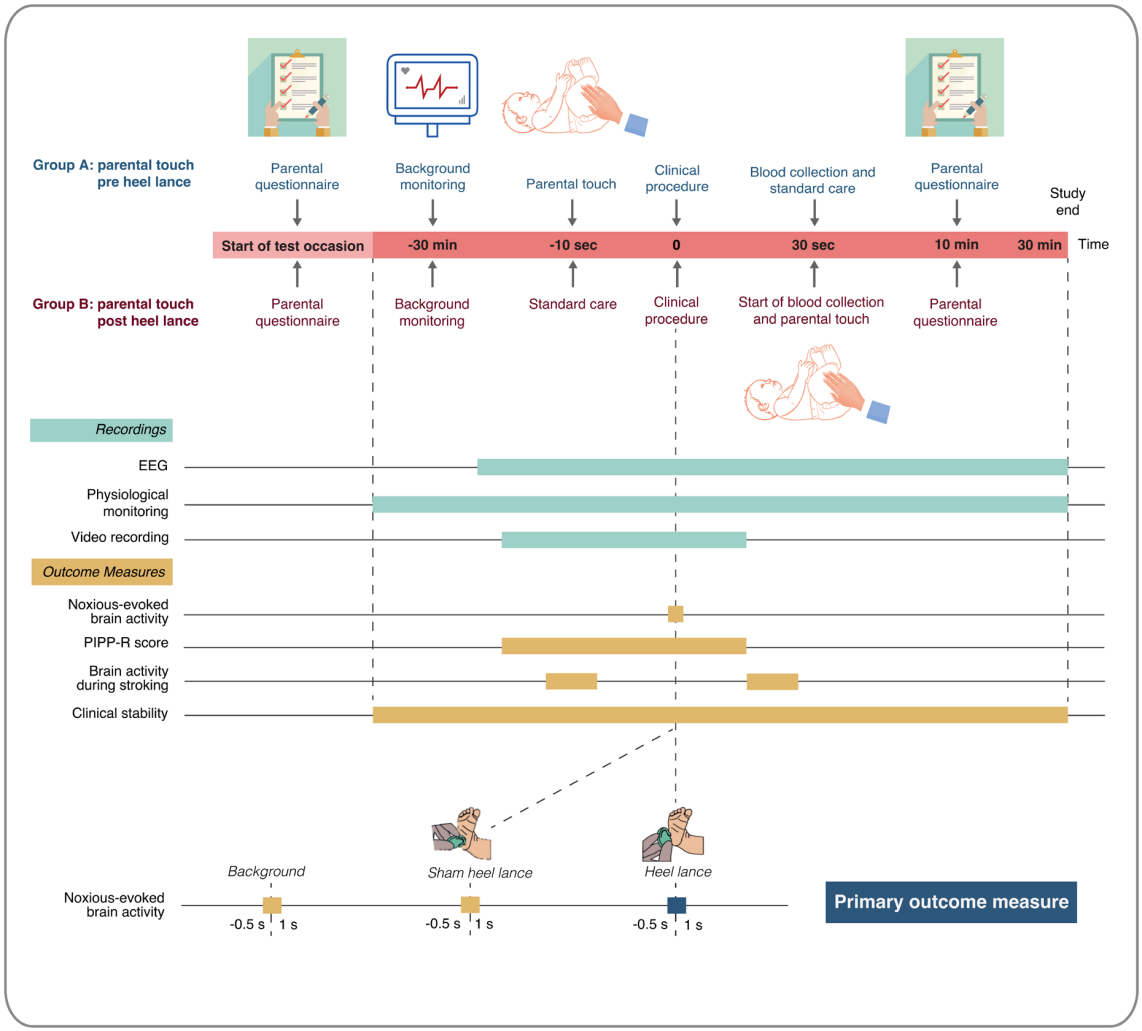
Timings of outcome recordings and outcome measures. Abbreviations: EEG = electroencephalography; PIPP-R = Premature Infant Pain Profile – Revised. This figure has been reproduced from our trial registration ( https://doi.org/10.1186/ISRCTN14135962) with permission from ISRCTN.

***Interim analyses and timings*.** No interim statistical analysis is planned. The timing of outcome assessment will vary depending on the outcome. An overview of the timings of outcome recordings and outcome measures for the Petal trial is presented in Figure 1.

### Statistical principles

***Confidence intervals and p-values*.** The level of statistical significance will be set at 5% for the primary outcome. The level of statistical significance for all secondary outcomes will be set at 5% overall. For secondary outcomes, multiple comparisons will be corrected with the Holm method. We will report effect sizes and 95% CI for all outcomes.

***Adherence and protocol deviations*.** Adherence to the intervention will be assessed qualitatively during the trial by observing the parent while stroking the infant. In the intervention group the protocol will be considered as adhered to if the start of the parental stroking commenced less than 45 seconds before the heel lance. Deviations from the assigned intervention will be reported alongside the results. If a participant is withdrawn from the study, the reason for withdrawal will be reported. We will report the timing of withdrawal in relation to communicating to parents their child’s allocation arm and in relation to the trial’s timeline wherever possible.

***Analysis populations*.** To assess the effects of parental stroking on relieving acute procedural pain in neonates, we will quantify the per protocol effect (also known as the effect of adhering to the intervention) ( Higgins *et al*., 2023), accounting for non-compliance in the analysis by performing complier average causal effect (CACE) analysis, which appropriately isolates the per protocol effect through instrumental variable analysis using the two-stage least squares approach ( Peugh *et al*., 2017). We will perform CACE analysis on the full analysis set, which includes all randomised patients with a measured outcome, and is thus as close as possible to the intention-to-treat ideal of including all randomised subjects ( ICH Expert Working Group, 1998). Parental questionnaire data will be included in the analysis irrespective of the timing of the stroking intervention.

### Trial population

**Figure 2.**
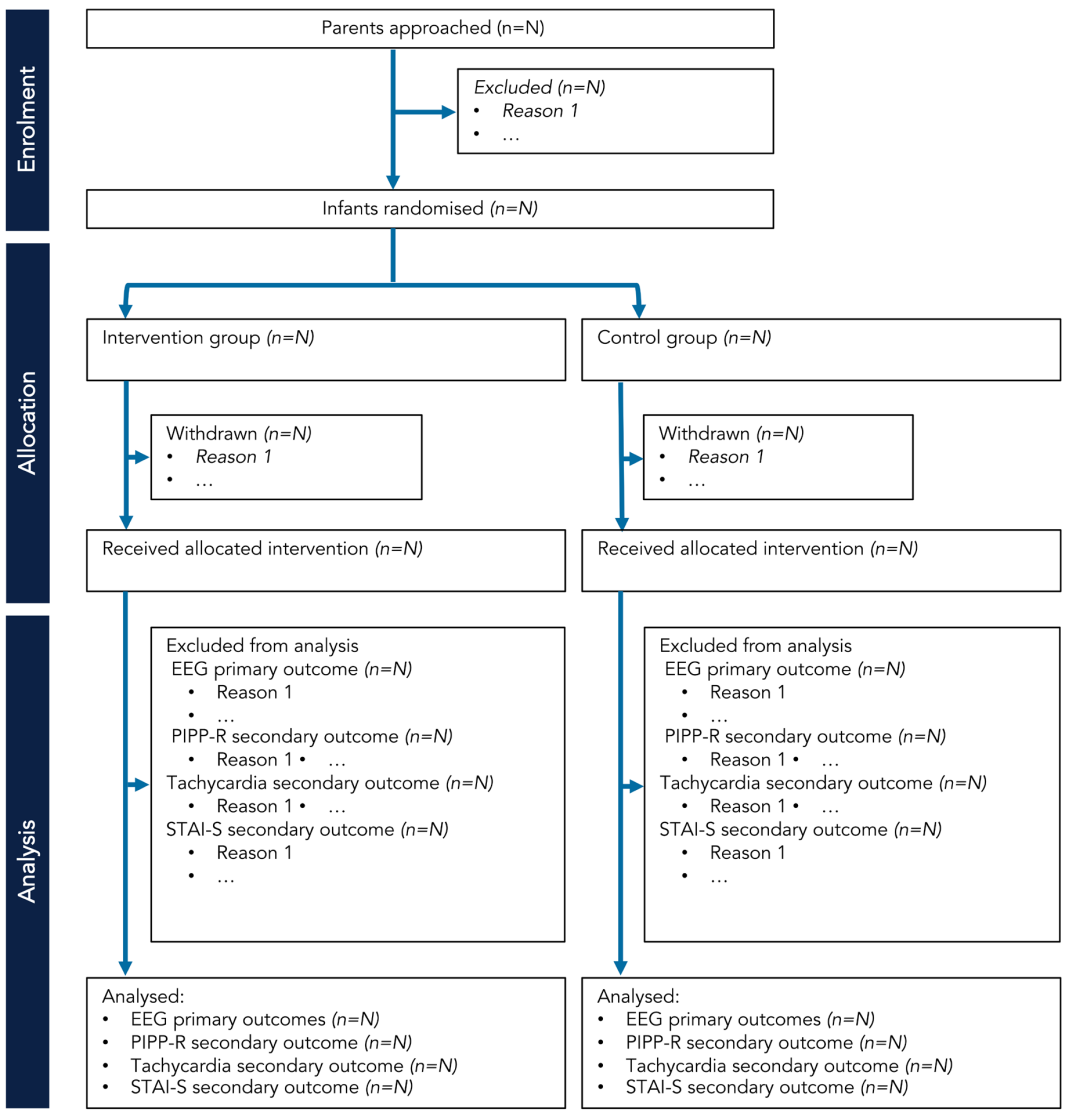
CONSORT flow diagram schematic for Petal trial. Abbreviations: EEG = electroencephalography; PIPP-R = Premature Infant Pain Profile – revised; STAI-S = State-Trait Anxiety Inventory – State.

***Screening, eligibility, recruitment, withdrawal*.** The eligibility criteria for the Petal trial are outlined in Table 2. The number of potential participants approached will be recorded and compared to the number of participants included in the trial. Parents may withdraw their neonate from the trial at any time and they are not obliged to give a reason. If parents choose to withdraw their child after the study has begun, they will be asked whether data already collected may be retained and used for the purposes of the trial. Parents will be made aware that this decision has no impact on any aspects of their infant’s continuing care. The attending clinician may withdraw the neonate from the trial if they consider this to be in the best interest of the neonate’s health and well-being. If any of the exclusion criteria manifest after consent but prior to data collection, the participant will be withdrawn. We will present the progress of participants through the phases of the trial (from enrolment to analysis) using the CONSORT flow diagram ( Schulz *et al*., 2010) – see schematic flow diagram in Figure 2.

**Table 2.** Eligibility criteria.

**Inclusion**	Participants born at the John Radcliffe Hospital, Oxford or the Royal Devon and Exeter Hospital, Devon
	Neonates born at or after 35+0 weeks gestation
	Neonates with a postnatal age of ≤7 days
	Neonates who require a heel lance as part of clinical care
	Neonates for whom parents/guardians have given written informed consent for participation
**Exclusion**	Hypoxic Ischaemic Encephalopathy
	Intraventricular haemorrhage (IVH) > grade II
	Received any analgesics or sedatives in the last 24 hours
	Congenital malformation or genetic condition known to affect neurological development
	Born to mothers who have a history of substance abuse.

***Baseline characteristics*.** We will report the following baseline patient characteristics: gestational age at birth (weeks); postmenstrual age at time of study (weeks); postnatal age at time of study (days); birthweight (g); sex; mode of delivery; Apgar score at 1 min; Apgar score at 5 min; primary reason for blood test; estimated cumulative prior pain exposure. Postmenstrual age at time of study is measured as the sum of gestational age and postnatal age, both of which will be recorded in the neonate’s medical notes. Cumulative pain exposure is measured from each neonate’s clinical records as the total number of acute skin-breaking procedures, including heel lances, venepuncture, and intravenous cannulations, and aspirations (oropharyngeal or endotracheal) from time of birth to time of study.

### Analysis

***Outcome definitions*.** The primary outcome measure is the magnitude of noxious stimulus-evoked brain activity following a heel lance: EEG data recorded in the 1000 ms post-lance period from the Cz-Fz channel, where Cz is the recording electrode and Fz is the reference electrode. After preprocessing, the n-NRF will be regressed onto the EEG data in the region 400–700 ms ± 100 ms. The magnitude of noxious stimulus-evoked brain activity is the n-NRF scaling factor i.e. the regression coefficient. Two experienced blinded investigators will visually review the EEG epochs for the presence of artefact. Any discrepancies in assessment will be resolved by discussion. Epochs will be rejected for the primary outcome measure analysis if they contain gross movement or electrical artefacts.

Clinical pain scores will be calculated using the PIPP-R ( Stevens *et al*., 2014). The score is composed of postmenstrual age, behavioural state, physiological changes (heart rate and oxygen saturation changes), as well as facial expression duration (brow bulge, eye squeeze and nasolabial furrow) in the 30 s post-procedure. The PIPP-R score is the sum of the ratings for each component of the assessment, and has a range of 0–21. Videos and vital signs will be used to calculate the behavioural, heart rate and oxygen saturation components of the PIPP-R score; scores are not calculated live. The behavioural (facial expressions) components of the PIPP-R will be scored by researchers who have not been involved in the specific test occasion and who were blind to the procedure (sham heel lance or heel lance) and trial arm allocation. PIPP-R vital signs components will be calculated using automated scripts on blinded data. Each PIPP-R video will be initially scored by an individual rater. Subsequently, 20% of videos will be re-scored, either by a different researcher for the purpose of assessing inter-rater reliability or by the researcher who did the initial scoring to assess intra-individual reliability. The non-video components for the PIPP-R ratings will be calculated automatically and reliability will be assessed on the total PIPP-R score. If reliability is high (0.9 or greater), the initial video scoring will be used; if reliability is low, scores will be averaged.

The tachycardia outcome per infant will be dichotomous: did the infant become tachycardic, yes or no. Tachycardia will be defined as an increase in heart rate over 160 bpm in the 30 s period post heel lance if the mean heart rate in the baseline period is less than 160 bpm. The baseline period is defined as the 15 s period prior to the start of the stroking.

If more than one heel lance is required during the blood test procedure, only the first heel lance will be used for calculating the three neonate’s outcomes: EEG, PIPP-R, tachycardia. Any subsequent heel lances will not occur within the 30 s after the first heel lance, as these 30 s are part of the response estimation.

The State-Trait Anxiety Inventory (STAI) is a widely used, validated and reliable assessment for anxiety ( Spielberger *et al*., 1983). The STAI-S scale (anxiety state) consists of 20 statements. It will require the parents to rate their anxiety at a particular moment in time (pre- and post-procedure for this trial) and is used to determine the parental level of anxiety induced by stressful procedures. Each question is rated on a 4-point Likert scale. STAI-S thus has a range of 20–80.

***Analysis methods*.** The primary outcome magnitude of noxious stimulus-evoked brain activity will be compared between the two groups using multiple linear regression analysis via the general linear model (GLM). In the GLM, we will control for the five minimisation variables. We will assess the relevant assumptions for our chosen statistical test. Given our design, model, and variables of interest, we do not expect issues with linearity, independence of residuals, or exogeneity of variables. We will assess the linearity assumption using multivariable fractional polynomials ( Royston & Altman, 1994); if any violation of independence of residuals occurs (e.g. recruitment of twins), we will use permutation testing with appropriate exchangeability blocks ( Winkler *et al*., 2014); and exogeneity is achieved by experimental design i.e. randomisation ( Hernan & Robins, 2024). For significant deviations from normality (assessed using Lilliefors test), we will consider linear regression via permutation testing. Permutation testing will be implemented using the PALM toolbox with 10,000 permutations, as described in the toolbox’s accompanying publication ( Winkler *et al*., 2014) – PALM allows specification of exchangeability blocks, for use in cases where independence is violated but exchangeability can be maintained in the analysis (e.g. twins). For significant deviations from homoscedasticity (assessed using Breusch Pagan test), we will consider a robust standard error linear regression. We will present the mean, SD, and 95% CI for the effect size of interest, i.e., the difference in mean magnitudes of noxious stimulus-evoked brain activity between groups.

PIPP-R scores will be compared between the two groups using multiple linear regression analysis. In the GLM, we will control for the five minimisation variables. As for the primary EEG outcome linear regression, we will test the relevant assumptions, choose the appropriate analysis, and report the statistical metrics, as outlined above.

Tachycardia will be compared between two groups using multiple logistic regression analysis via a generalized linear regression model with binomial distribution and logit link function. In this model, we will control for the five minimisation variables. We will assess the relevant assumptions for our chosen statistical test. Given our design, model, and variables of interest, we do not expect issues with linearity between log odds ratio and explanatory variables, independence of residuals, or exogeneity of variables – see explanation above for primary outcome. We will further ensure that we have no perfect separation. We will present the odds ratio with a 95% CI.

The STAI-S scores will be compared between two groups using multiple linear regression analysis. In the GLM, we will control for the five minimisation variables and a baseline estimate of pre-procedural anxiety (pre-procedural STAI-S). As for the primary EEG outcome and secondary PIPP-R outcome linear regressions, we will test the relevant assumptions, choose the appropriate analysis, and report the statistical metrics, as outlined above.

Researchers involved in the test occasion could not be blinded to the group allocation due to the nature of the study. Researchers who performed subjective outcome assessment of the EEG data (primary outcome measure), PIPP-R components and tachycardia outcome (secondary outcomes) were blinded to the group allocation of the participant whose outcome they were assessing. Further details on blinding for each outcome are included in the full SAP ( https://doi.org/10.1186/ISRCTN14135962).

***Missing data*.** Missing data will occur in our trial due to, for example, equipment failure, artefacts within the EEG recording, or clinical care changes if, for example, the heel lance is not deemed necessary anymore by the clinical team after a participant has already been enrolled. For both primary and secondary outcomes, we will assess the impact of missingness as follows:

1. Describe the reasons for missing outcomes, wherever possible.
2. Describe the extent of missingness by trial arm.
3. Describe the extent of missingness by covariates that might influence the missing variable or the pattern of missingness.

For the primary outcome only, we will do the following sensitivity analyses:

1. Multiple imputation to assess sensitivity to data missing at random.
2. Pattern mixture model to assess sensitivity to data missing not at random.

***Additional analyses*.** We will assess and report intra- and inter-rater reliability for the PIPP-R ratings. We will use the intraclass correlation coefficient and a one-way model to assess rater consistency. Reliability will be assessed on the total PIPP-R score only.

***Harms*.** A summary of safety data including severe adverse events causally linked to trial participation will be included in the results.

***Statistical software*.** Primary and secondary analyses will be performed using MATLAB (version 9.14, R2023a); missing data analyses and additional analyses will be performed using R (version 4.2.2).

## Discussion

The present SAP outlines the analysis plan for a trial that uses a brain imaging outcome for neonatal pain assessment. Paediatric pain trials have been highlighted by the US Food and Drug Administration (FDA) as an important and challenging topic ( Hertz, 2016), and recently the FDA have hosted a workshop on the use of brain imaging in clinical trials for infant pain ( Slater, 2021). The Petal trial, to which this SAP relates, is part of a larger effort of establishing our brain-based EEG outcome measure of infant acute somatic nociceptive pain for use in clinical trials. Further, we are conducting an additional study in parallel to establish the measurement properties and interpretability of the EEG outcome measure, in collaboration with multiple regulatory bodies (e.g. FDA, EMA) and pharmaceutical companies (e.g. Pfizer, Sanofi) ( Baxter *et al*., 2023). The successful use of this specific EEG outcome measure in the present neonatal clinical pain trial to assess non-pharmacological analgesic efficacy will be an important milestone in establishing a brain-based pain indicator for future pharmacological and non-pharmacological analgesic efficacy trials. This SAP is thus likely to be of importance and interest to a broad research community, including those in academia, pharmaceutical companies, and regulatory bodies. We hope this SAP will help establish a foundation for the use of brain imaging outcomes in neonatal pain clinical trials on which best practices can be built.

## Data Availability

No data are associated with this article. An extended version of this SAP, which was registered prior to hard locking the database and prior to unblinding, is publicly available on the ISRCTN registry: https://doi.org/10.1186/ISRCTN14135962 ( Slater, no date).
