# Supplementary material for: Statistical analysis plan for the Petal trial: the effects of parental touch on relieving acute procedural pain in neonates
Source: Wellcome Open Res. 2024 Dec 18;8:402. Originally published 2023 Sep 15. [Version 3] doi: 10.12688/wellcomeopenres.19819.3 (PMC11920693; doi:10.12688/wellcomeopenres.19819.3)
Supplement: Peer review history

# Reviewer report: Reviewer response for version 3

**DOI:** 10.21956/wellcomeopenres.25969.r119415

**Published:** 2025-03-19

**Authors:** Mia Mclean

**Affiliations:** 1 Auckland University of Technology, Auckland, New Zealand

The article presents the Statistical Analysis Plan (SAP) for the Petal trial, a RCT comparing standard of care and parental stroking performed before a heel lance on neonates' noxious stimulus-evoked brain activity (primary outcome) and behavioural and physiological pain scores as well as parent anxiety (secondary outcomes). 

 The authors have presented a detailed SAP, and I look forward to reading the results of this important trial.

 The sham heel-lance is referenced with respect to the coding of the PIPP-R, it does not appear in any of the outcome measures. This is confusing; please clarify or remove from text.  

 Justification for GLM instead of MLM accounting for study site as cluster variable is needed for all outcomes. 

 Given pre-procedure parent anxiety is included in the GLM model, the outcome will represent "change in parent anxiety" from pre to post-procedure. 

 Suggest listing the minimisation variables again in Analysis Plan when first mentioned to clarify for reader the proposed model.

 This statement should be supported by a reference, "it is a pertinent and accessible feature of central importance to understanding neonates’ neural responses to noxious input and the neurophysiology of the early developing pain system." given that the Discussion details, "Further, we are conducting an additional study in parallel to establish the measurement properties and interpretability of the EEG outcome measure," and "The successful use of this specific EEG outcome measure in the present neonatal clinical pain trial to assess non-pharmacological analgesic efficacy will be an important milestone in establishing a brain-based pain indicator for future pharmacological and non-pharmacological analgesic efficacy trials.". Thus the initial statement seems in contrast to the Discussion.

Is the work clearly and accurately presented and does it cite the current literature?

Partly

If applicable, is the statistical analysis and its interpretation appropriate?

Partly

Are all the source data underlying the results available to ensure full reproducibility?

No source data required

Is the study design appropriate and is the work technically sound?

Yes

Are the conclusions drawn adequately supported by the results?

Yes

Are sufficient details of methods and analysis provided to allow replication by others?

Yes

Reviewer Expertise:

neonatal pain, preterm birth, perinatal stress, child development.

I confirm that I have read this submission and believe that I have an appropriate level of expertise to confirm that it is of an acceptable scientific standard, however I have significant reservations, as outlined above.

# Reviewer report: Reviewer response for version 3

**DOI:** 10.21956/wellcomeopenres.25969.r118417

**Published:** 2025-03-18

**Authors:** Carol McNair

**Affiliations:** 1 University of Toronto, Toronto, Ontario, Canada

The changes to the SAP have made the SAP stronger and the authors approach is sound. The authors have clearly described their methodology and plan for analysis.  I will await the findings of their study with interest as this area of EEG response to pain and pain management is quite novel and will be of great interest to those interested in infant pain managment

Is the work clearly and accurately presented and does it cite the current literature?

Yes

If applicable, is the statistical analysis and its interpretation appropriate?

Yes

Are all the source data underlying the results available to ensure full reproducibility?

Yes

Is the study design appropriate and is the work technically sound?

Yes

Are the conclusions drawn adequately supported by the results?

Yes

Are sufficient details of methods and analysis provided to allow replication by others?

Yes

Reviewer Expertise:

Neonatal pain assessment and management

I confirm that I have read this submission and believe that I have an appropriate level of expertise to confirm that it is of an acceptable scientific standard.

# Reviewer report: Reviewer response for version 2

**DOI:** 10.21956/wellcomeopenres.25618.r110132

**Published:** 2024-12-02

**Authors:** Lorenzo Fabrizi

**Affiliations:** 1 University College London, London, England, UK

In this paper Baxter et al. draw the statistical analysis plan to evaluate the effect of stroking on various noxious-related responses in neonates. This effort is very laudable to reduce bias and increase reproducibility in the data analysis. The paper is well written and easy to follow. However, I would like the authors to revisit their interpretation of the outcome. The primary objective of the paper is specific and very well stated in Table 1: "To test whether parent touch prior to a clinically required heel lance reduces noxious stimulus-evoked brain activity", however throughout the text, title and abstract this becomes "analgesia" and reference to "pain reduction", "pain relieving" or "pain indicator" often appears. This is an interpretation of the evidence. These statements should be replaced with more accurate references to modulation of noxious-stimulus evoked activity. Especially considering how complicated the pain experience is. For example the title should be re-written as "the effect of parental stroking on modulating noxious-related activity in neonates". The potential interpretation that this could represent analgesia should be part of the discussions, where the caveat of this interpretation should also be mentioned. Also "parental touch" should be replaced with "parental stroking" throughout for precision. Also, there is no direct evidence that stroking at any speed activates neonates' C-tactile (CT) fibre, so the statement regarding this in the introduction should be rephrased to "This intervention may reduce the cortical response to a noxious stimulus".

**The description of the stats is overall clear, but there is a few points that need clarifications. Here are some comments:**

- The expect reduction of 40% is fundamental in defining the sample size. A more detail explanation using data from the cited publication is needed to justify this number.
- Can you expand on this statement: "we do not expect issues with linearity, independence of residuals or exogeneity of variables"? Why is that?
- What does "the group allocation variable will be adjusted for the five minimization variables"? How can you adjust a variable? Do you mean that you will adjust the effect of the group allocation variable with the other minimization variables? You should rewrite as: "The analysis will control for the five minimization variables when assessing the effect of the group allocation variable."|
- Can you please write down the model you intend to use to avoid misunderstandings?
- What does "we will consider linear regression via permutation testing" mean? Do you mean that you will test the null hypothesis that the model parameters are = 0 with permutation testing? How many permutation will you perform? What will you be permuting? This needs to be clearer. Also can you consolidate this part of the analysis methods with the objective section where the you state the null and alternative hypothesis? Use the same notation throughout.
- For Tachycardia and STAI-S scores what are the assumptions? It cannot be that you are expecting a normal distribution. Can you please specify them? How will the odd ratios for tachycardia be calculated? And the confidence intervals? What are you going to report for STAI-S scores?

**Another point:**

- What will be the use of the sham heel lance? It does not appear in any of the outcome measures.

Is the work clearly and accurately presented and does it cite the current literature?

Partly

If applicable, is the statistical analysis and its interpretation appropriate?

Partly

Are all the source data underlying the results available to ensure full reproducibility?

No source data required

Is the study design appropriate and is the work technically sound?

Yes

Are the conclusions drawn adequately supported by the results?

Partly

Are sufficient details of methods and analysis provided to allow replication by others?

Partly

Reviewer Expertise:

Neonatal brain development, neonatal EEG, neonatal nociception, neonatal brain imaging

I confirm that I have read this submission and believe that I have an appropriate level of expertise to confirm that it is of an acceptable scientific standard, however I have significant reservations, as outlined above.

# Response

**Published:** 2024-12-16

**Authors:** Luke Baxter

**Affiliations:** Department of Paediatrics, University of Oxford, Oxford, England, UK

In this paper Baxter et al. draw the statistical analysis plan to evaluate the effect of stroking on various noxious-related responses in neonates. This effort is very laudable to reduce bias and increase reproducibility in the data analysis. The paper is well written and easy to follow.  

**AUTHOR RESPONSE: **Thank you very much for your positive review and helpful feedback. The edits we have made to the manuscript consequently have helped improve the overall quality of the paper. 

 However, I would like the authors to revisit their interpretation of the outcome. The primary objective of the paper is specific and very well stated in Table 1: "To test whether parent touch prior to a clinically required heel lance reduces noxious stimulus-evoked brain activity", however throughout the text, title and abstract this becomes "analgesia" and reference to "pain reduction", "pain relieving" or "pain indicator" often appears. This is an interpretation of the evidence. These statements should be replaced with more accurate references to modulation of noxious-stimulus evoked activity. Especially considering how complicated the pain experience is. For example the title should be re-written as "the effect of parental stroking on modulating noxious-related activity in neonates". The potential interpretation that this could represent analgesia should be part of the discussions, where the caveat of this interpretation should also be mentioned.  

**AUTHOR RESPONSE:  ** We agree that pain is a complex phenomenon and that there is room for misinterpretation and overinterpretation with terminology. We have thus added a detailed paragraph early in the introduction to clarify and add appropriate information for the reader. We retain the use of the word “pain” throughout to maintain consistency with the use of pain terminology in the field. We include this rationale in our newly included text: *“Due to the subjective nature of pain and the non-verbal nature of neonates, there is no gold standard pain measure for this population. While our primary outcome (the n-NRF magnitude) neither directly reflects nociception, the neural process of encoding noxious stimuli (IASP, 2024; Raja et al., 2020), nor pain experience, the unpleasant sensory and emotional subjective experience (IASP, 2024; Raja et al., 2020), it is a pertinent and accessible feature of central importance to understanding neonates’ neural responses to noxious input and the neurophysiology of the early developing pain system. Additionally, the n-NRF magnitude is a validated and reproducible pain-relevant measure (Aspbury et al., 2024; Hartley et al., 2017) that has demonstrated responsiveness to analgesic intervention (Cobo et al., 2021). With these considerations in mind, for consistency with established use of terminology, we refer to the n-NRF magnitude as a pain outcome throughout. This is consistent with pain terminology used in the field of neonatal pain assessment, for brain-based measures in both observational studies (Olsson et al., 2016; Rupawala et al., 2023) and clinical trials (Cornelissen, 2016; Wang, 2020).”* 

 Also "parental touch" should be replaced with "parental stroking" throughout for precision. **AUTHOR RESPONSE: **  We have updated the text to clarify that the touch is stroking. The first instance is maintained until it is clarified (in the same paragraph) that the “parental touch” is specifically “parental stroking”. 

 Also, there is no direct evidence that stroking at any speed activates neonates' C-tactile (CT) fibre, so the statement regarding this in the introduction should be rephrased to "This intervention may reduce the cortical response to a noxious stimulus".  

**AUTHOR RESPONSE :** We have rephrased this sentence to clarify that the suggested mechanism is only a potential mechanism of action. We must retain the mentioning of CT fibres, as it is the hypothesis that underpins the nature of the intervention i.e. stroking at a particular speed. *“This intervention may reduce noxious input reaching the brain, potentially through activating neonates' C-tactile (CT) fibre system ( Gursul et al., 2018; Liljencrantz et al., 2017; von Mohr et al., 2018).”*  

**The description of the stats is overall clear, but there is a few points that need clarifications. Here are some comments:**

- The expect reduction of 40% is fundamental in defining the sample size. A more detail explanation using data from the cited publication is needed to justify this number. 
**AUTHOR RESPONSE : **  We have added additional details about the expected 40% reduction in noxious-evoked brain activity in the intervention group, drawing on evidence from the cited references. *“In neonates who received soft brushing of the skin before the heel lance procedure, Gursul and colleagues (Gursul et al., 2018) observed a 40% reduction, and Cobo and colleagues (Cobo et al., 2021) observed a 39% reduction, in heel lance-evoked brain activity. In adult studies, similar reductions in noxious-evoked brain activity are accompanied by significantly lower verbal pain scores ( Lorenz et al., 1997; von Mohr et al., 2018).  Reductions of 30%-36% in pain intensity ratings are considered clinically "meaningful" as reported by a consensus statement for adult clinical pain trials (Dworkin et al., 2008). Therefore, we consider a 40% reduction in the intervention group to be a clinically significant outcome in this trial.”*  
- Can you expand on this statement: "we do not expect issues with linearity, independence of residuals or exogeneity of variables"? Why is that?
**AUTHOR RESPONSE: **  We have added text to the “Analysis methods” section to clarify these points. *“We will assess the linearity assumption using multivariable fractional polynomials (Royston and Altman, 1994); if any violation of independence of residuals occurs (e.g. recruitment of twins), we will use permutation testing with appropriate exchangeability blocks (Winkler et al., 2014); and exogeneity is achieved by experimental design i.e. randomisation (Hernan and Robins, 2024).”*   
- What does "the group allocation variable will be adjusted for the five minimization variables"? How can you adjust a variable? Do you mean that you will adjust the effect of the group allocation variable with the other minimization variables? You should rewrite as: "The analysis will control for the five minimization variables when assessing the effect of the group allocation variable."
**AUTHOR RESPONSE: ** Thank you for spotting the poor wording. Yes, the association will be controlled for the five minimisation variables. We have updated the wording accordingly.  
- Can you please write down the model you intend to use to avoid misunderstandings?
**AUTHOR RESPONSE :**   For the n-NRF magnitude, PIPP-R score, and STAI-S score, the model is the general linear model. For the tachycardia outcome, the model is the generalised linear model with binomial distribution and logit link function. We have added these details to the relevant sentences in the “Analysis methods” section for each outcome.   

- What does "we will consider linear regression via permutation testing" mean? Do you mean that you will test the null hypothesis that the model parameters are = 0 with permutation testing? How many permutation will you perform? What will you be permuting? This needs to be clearer. Also can you consolidate this part of the analysis methods with the objective section where the you state the null and alternative hypothesis? Use the same notation throughout.
** AUTHOR RESPONSE:**  The details of permutation approach for the GLM are detailed in the Winkler et al 2014 paper, which we now reference in the SAP. The PALM toolbox that implements the methods detailed in this paper will be used. We will use 10,000 permutations. The null and alternative hypothesis remain unchanged from that specified in the Introduction. The only difference is the permutation approach builds an empirical distribution for the test statistic, rather than relying on parametric assumptions of the distribution. We have added additional detail to the manuscript. *“Permutation testing will be implemented using the PALM toolbox with 10,000 permutations, as described in the toolbox’s accompanying publication (Winkler et al., 2014) – PALM allows specification of exchangeability blocks, for use in cases where independence is violated but exchangeability can be maintained in the analysis (e.g. twins).”* 
- For Tachycardia and STAI-S scores what are the assumptions? It cannot be that you are expecting a normal distribution. Can you please specify them? How will the odd ratios for tachycardia be calculated? And the confidence intervals? What are you going to report for STAI-S scores?
**AUTHOR RESPONSE :** Based on the request for clarification above, we have now specified the statistical model per outcome (general linear model for n-NRF, PIPP-R, and STAI-S; and generalised linear model for tachycardia). It should now hopefully be clear that the assumptions for STAI-S are the same as that for the primary outcome, given they are both analysed using the same model. The assumptions are: linearity, exogeneity, independence, normality, and homoscedasticity. For tachycardia, we have added further details about the model: “with binomial distribution and logit link function”. The assumptions are: linearity, exogeneity, and independence. Thus, only STAI-S assumes normality. Normality will be tested, and if found to be violated, will be addressed by using permutation testing, which removes the distribution assumption by instead using an empirical distribution.   Odds ratios are derived from the generalised linear model in the standard way i.e. exponential of regression beta.   Confidence intervals are derived from the general and generalised linear models in the standard way. We are using MATLAB functions fitlm and fitglm. In both cases, confidence intervals are derived using coefCI function.   For STAI-S, we will report the same outputs as per the primary outcome. Given the primary outcome, PIPP-R, and STAI-S all use the same model (general linear model), the reported metrics will be consistent across these. Given we have now clarified that the same model is used across these three outcomes, we hope it is now clear that the reported metrics should also be the same.   
**Another point:**
- What will be the use of the sham heel lance? It does not appear in any of the outcome measures.
**AUTHOR RESPONSE: **  The sham heel lance is collected only for a preliminary data quality assessment as a positive control. Given the heel lance is a noxious skin-breaking stimulus and the sham heel lance is non-noxious and non-skin-breaking, a pain-relevant metric (e.g. n-NRF magnitude, PIPP-R score) should have a larger response to the heel lance compared to the sham lance. Given this QC assessment is an initial sanity check of basic data quality, we have not added these details to the SAP for reasons of flow and brevity.  

# Reviewer report: Reviewer response for version 1

**DOI:** 10.21956/wellcomeopenres.21951.r70684

**Published:** 2024-04-02

**Authors:** Anna Heath, Arlene Jiang

**Affiliations:** 1 The Hospital for Sick Children, University of Toronto, Toronto, Canada; 2 University College London, London, England, UK; 3 Child Health Evaluative Sciences, The Hospital for Sick Children, Toronto, Ontario, Canada

Review:  

 Statistical analysis plan for the Petal trial: the effects of parental touch on relieving acute procedural pain in neonates 

 General Remarks 

 Baxter et al. outline a statistical analysis plan for the Petal trial, which evaluates the effect of parental touch on treating procedural pain in neonates. The trial compares pain-related outcomes when parental touch is applied before vs. after a heel lance, with a primary outcome of magnitude of a noxious stimulus-evoked brain activity. This SAP (Statistical Analysis Plan) aims to improve the reproducibility of the Petal study results and are based on best practice guidelines for SAP development. The authors are thorough in covering the 55 items specified in the JAMA guidelines by Gamble et al.  

 We have identified a range of suggestions to further enhance reproducibility, replicability and readability of the SAP.  

 Major Comments 

- Analysis populations: The authors state that “we will quantify the per protocol effect”. A per protocol population typically diminishes the comparability of the randomized groups. To ascertain a causal effect of an intervention, an intention-to-treat approach is typically suggested. Could an intention-to-treat approach be reported here, with a per protocol sensitivity analysis? Additionally, the definition of the “per protocol” population is not clear as the SAP suggests that non-compliance will be “accounted for” in the analysis. If the authors continue with their proposed analysis, can they clarify what is meant by “account for”? 
- Analysis – Outcome definitions: The authors mention “After preprocessing, the n-NRF…”, but do not provide any other information about preprocessing here. I see that there is further information on preprocessing in the ISRCTN SAP. In the interest of transparent statistical conduct, I would recommend including information on the “subjective assessment” of artefact detection/rejection in this publication. I would also suggest that the authors provide descriptive definitions for artefact items commonly rejected.  
- Analysis – Outcome definitions: Could you clarify how many channels you are analyzing as well as what reference channel you are using for the primary outcome? This is also not clear.  
- Analysis – Outcome definitions: The authors provide information on the scoring system for the PIPP-R scale, but can they clarify who will be measuring this outcome? How many individuals? Will they be scoring live, or will the procedure be recorded? 
- Analysis Methods: We appreciate that the authors have specified that they will test the assumptions of their different tests. However, there is a lack of detail on how these assumptions will be tested. Will you use statistical tests? Will you use graphical representations? What alternative analysis methods would you use if these assumptions are not valid? 

Specific Comments  

- Abstract/Introduction/Extended data: There are a few mentions of “unblinding” throughout the manuscript. Could you explicitly mention who (i.e., analysts, outcome assessors) is blinded?  
- Methods – Study Design: You mention “standard of care” in this section. Could you explicitly define standard of care?  
- Randomization: Can you clarify why the minimization algorithm was chosen and why the minimization variables were selected? 
- Sample size: Can the authors clarify the statistical model assumed for the sample size calculation? From our reading, it seems a t-test was used but this should be clarified, given that the primary analysis is adjusted. 
- Baseline characteristics: How are (i) postmenstrual age at time of study (ii) cumulative pain exposure measured? 
- Missing Data: Can you clarify whether you are planning to impute the outcome data? Or impute the covariates for the primary outcome model? It is not standard practice to impute outcome data. 
- Additional analyses: The authors mention “intra- and inter-rater reliability” here. Could you clarify how PIPP-R scores from different raters will be used (i.e., averaged?) in the Outcome definitions section, as well as the number of raters? 

Is the work clearly and accurately presented and does it cite the current literature?

Yes

If applicable, is the statistical analysis and its interpretation appropriate?

Yes

Are all the source data underlying the results available to ensure full reproducibility?

No source data required

Is the study design appropriate and is the work technically sound?

Yes

Are the conclusions drawn adequately supported by the results?

Yes

Are sufficient details of methods and analysis provided to allow replication by others?

Partly

Reviewer Expertise:

Statistical Methods, Clinical Trials, Procedural Pain, Health Economic Modelling.

We confirm that we have read this submission and believe that we have an appropriate level of expertise to confirm that it is of an acceptable scientific standard, however we have significant reservations, as outlined above.

# Response

**Published:** 2024-10-08

**Authors:** Luke Baxter

**Affiliations:** Department of Paediatrics, University of Oxford, Oxford, England, UK

Review:

 Statistical analysis plan for the Petal trial: the effects of parental touch on relieving acute procedural pain in neonates

 General Remarks Baxter et al. outline a statistical analysis plan for the Petal trial, which evaluates the effect of parental touch on treating procedural pain in neonates. The trial compares pain-related outcomes when parental touch is applied before vs. after a heel lance, with a primary outcome of magnitude of a noxious stimulus-evoked brain activity. This SAP (Statistical Analysis Plan) aims to improve the reproducibility of the Petal study results and are based on best practice guidelines for SAP development. The authors are thorough in covering the 55 items specified in the JAMA guidelines by Gamble et al.

 We have identified a range of suggestions to further enhance reproducibility, replicability and readability of the SAP.  

 Major Comments

- Analysis populations: The authors state that “we will quantify the per protocol effect”. A per protocol population typically diminishes the comparability of the randomized groups. To ascertain a causal effect of an intervention, an intention-to-treat approach is typically suggested. Could an intention-to-treat approach be reported here, with a per protocol sensitivity analysis? Additionally, the definition of the “per protocol” population is not clear as the SAP suggests that non-compliance will be “accounted for” in the analysis. If the authors continue with their proposed analysis, can they clarify what is meant by “account for”? **Response: **We realise the terminology surrounding per protocol and ITT is ambiguous and inconsistent in the literature. In this manuscript, we use the terminology used in the *Cochrane Handbook for Systematic Reviews of Interventions*, which is freely available online ( https://training.cochrane.org/handbook/current). The definition of the “per protocol effect” is provided in chapter 8 section 8.2.2 and is also referred to as the “effect of adhering to the intervention”, while the “intention-to-treat” effect is also referred to as the “effect of assignment to the intervention”. Here are the definitions from the relevant section: *“1. the effect of **assignment** to the interventions at baseline, regardless of whether the interventions are received as intended (the ‘intention-to-treat effect’); or* *2. the effect of **adhering to** the interventions as specified in the trial protocol (the ‘per-protocol effect’) (Hernán and Robins 2017).”*  
  We are primarily interested in the “per protocol effect” because:
  “ *Patients and other stakeholders are often interested in the effect of **adhering to **the intervention ****as described in the trial protocol (the ‘per-protocol effect’), because it relates most closely to the implications of their choice between the interventions.*”.  
  As outlined in the handbook, there are different ways to calculate the per protocol effect, with ‘as-treated’ analyses and naïve ‘per-protocol’ analyses being inappropriate. To correctly account for non-compliance, instrumental variable approaches are appropriate:
*“For the effect of adhering to intervention, appropriate analysis approaches are described by Hernán and Robins (Hernán and Robins 2017). Instrumental variable approaches can be used in some circumstances to estimate the effect of intervention among participants who received the assigned intervention.”*  
  We will calculate the per protocol effect appropriately using the instrumental variable approach called Complier Average Causal Effect (CACE) analysis, which is a two-stage least squares approach, and which allows for causal inference (Peugh JL, Strotman D, McGrady M, Rausch J, Kashikar-Zuck S. Beyond intent to treat (ITT): a complier average causal effect (CACE) estimation primer. J Sch Psychol 2017; 60: 7–24.)  
  Importantly, the “per protocol effect” and the “intention to treat effect” are different conceptually and both interesting and meaningful in their own rite and are thus not sensitivity analyses for each other. For completeness, we will also report the intention to treat effect as an exploratory outcome.  
  For both the primary per protocol effect analysis and the exploratory intention to treat effect, we will use the full analysis set, as defined in ICH E9 section 5.2.1. ( https://database.ich.org/sites/default/files/E9_Guideline.pdf):
*“In this document the term 'full analysis set' is used to describe the analysis set which is as complete as possible and as close as possible to the intention-to-treat ideal of including all randomised subjects”*  
  We have updated the manuscript to clarify all the above details as concisely as possible (see section ‘Analysis populations’).  
*“To assess the effects of parental touch on relieving acute procedural pain in neonates, we will quantify the per protocol effect (also known as the effect of adhering to the intervention) (Higgins et al., 2023), accounting for non-compliance in the analysis by performing complier average causal effect (CACE) analysis which appropriately isolates the per protocol effect through instrumental variable analysis using the two-stage least squares approach (Peugh et al., 2017). We will perform CACE analysis on the full analysis set, which includes all randomised patients with a measured outcome, and is thus as close as possible to the intention-to-treat ideal of including all randomised subjects (ICH Expert Working Group, 1998). Parental questionnaire data will be included in the analysis irrespective of the timing of the stroking intervention.”*  
- Analysis – Outcome definitions: The authors mention “After preprocessing, the n-NRF…”, but do not provide any other information about preprocessing here. I see that there is further information on preprocessing in the ISRCTN SAP. In the interest of transparent statistical conduct, I would recommend including information on the “subjective assessment” of artefact detection/rejection in this publication. I would also suggest that the authors provide descriptive definitions for artefact items commonly rejected.
**Response: **We have added these details to the manuscript (see section ‘Outcome definitions’).  
*“Two experienced blinded investigators will visually review the EEG epochs for the presence of artefact. Any discrepancies in assessment will be resolved by discussion. Epochs will be rejected for the primary outcome measure analysis if they contain gross movement or electrical artefacts.”*    
- Analysis – Outcome definitions: Could you clarify how many channels you are analyzing as well as what reference channel you are using for the primary outcome? This is also not clear.
**Response: **We have updated the manuscript to include these details (see section ‘Outcome definitions’).  
*“The primary outcome measure is the magnitude of noxious stimulus-evoked brain activity following a heel lance: EEG data recorded in the 1000 ms post-lance period from the Cz-Fz channel, where Cz is the recording electrode and Fz is the reference electrode.”*  
- Analysis – Outcome definitions: The authors provide information on the scoring system for the PIPP-R scale, but can they clarify who will be measuring this outcome? How many individuals? Will they be scoring live, or will the procedure be recorded?
**Response: **We have updated the manuscript to include these details (see section ‘Outcome definitions’).  
*“Clinical pain scores will be calculated using the PIPP-R (Stevens et al., 2014). The score is composed of postmenstrual age, behavioural state, physiological changes (heart rate and oxygen saturation changes), as well as facial expression duration (brow bulge, eye squeeze and nasolabial furrow) in the 30 s post-procedure. The PIPP-R score is the sum of the ratings for each component of the assessment and has a range of 0–21. Videos and vital signs will be used to calculate the behavioural, heart rate and oxygen saturation components of the PIPP-R score; scores are not calculated live. The behavioural (facial expressions) components of the PIPP-R will be scored by researchers who have not been involved in the specific test occasion and who were blind to the procedure (sham heel lance or heel lance) and trial arm allocation. PIPP-R vital signs components will be calculated using automated scripts on blinded data. Each PIPP-R video will be initially scored by an individual rater. Subsequently, 20% of videos will be re-scored, either by a different researcher for the purpose of assessing inter-rater reliability or by the researcher who did the initial scoring to assess intra-individual reliability. The non-video components for the PIPP-R ratings will be calculated automatically and reliability will be assessed on the total PIPP-R score. If reliability is high (0.9 or greater), the initial video scoring will be used; if reliability is low, scores will be averaged.*”  
- Analysis Methods: We appreciate that the authors have specified that they will test the assumptions of their different tests. However, there is a lack of detail on how these assumptions will be tested. Will you use statistical tests? Will you use graphical representations? What alternative analysis methods would you use if these assumptions are not valid?
   
**Response: **We have updated the manuscript to include these details (see section ‘Analysis methods’).  
*“For significant deviations from normality (assessed using Lilliefors test), we will consider linear regression via permutation testing. For significant deviations from homoscedasticity (assessed using Breusch Pagan test), we will consider a robust standard error linear regression.”*  
  Specific Comments
- Abstract/Introduction/Extended data: There are a few mentions of “unblinding” throughout the manuscript. Could you explicitly mention who (i.e., analysts, outcome assessors) is blinded?
   
**Response: **We have updated the manuscript to include these details (see section ‘Analysis methods’):  
*“Researchers involved in the test occasion could not be blinded to the group allocation due to the nature of the study. Researchers who performed subjective outcome assessment of the EEG data (primary outcome measure), PIPP-R components and tachycardia outcome (secondary outcomes) were blinded to the group allocation of the participant whose outcome they were assessing. Further details on blinding for each outcome are included in the full SAP (* *https://doi.org/10.1186/ISRCTN14135962* *).”*  
- Methods – Study Design: You mention “standard of care” in this section. Could you explicitly define standard of care?
**Response: **We have added text to detail standard of care in each research site (see section ‘Trial design and framework’).  
*“In both sites, standard of care involves offering all neonates comfort measures including swaddling and non-nutritive sucking, in line with local practice guidelines.”*  
- Randomization: Can you clarify why the minimization algorithm was chosen and why the minimization variables were selected?
**Response: **We have updated the manuscript to include these details (see section ‘Randomisation’).  
*“The randomisation program will use a minimisation algorithm to ensure approximate balance between the groups with respect to five minimisation variables that are likely to cause meaningful variability in pain response estimates: gestational age at birth, postnatal age at time of randomisation, sex, the primary indication for blood sampling, and research site. The minimisation approach was chosen due to its capacity to balance multiple prognostic variables in trials with small sample sizes, which would unlikely achieve balance through pure randomisation approaches.”*  
- Sample size: Can the authors clarify the statistical model assumed for the sample size calculation? From our reading, it seems a t-test was used but this should be clarified, given that the primary analysis is adjusted.
**Response: **We have updated the manuscript to clarify the statistical model assumed for the sample size calculation, which was a t-test (see section ‘Sample size’).  
*“Assuming a t-distribution, with 90% power, a two-sided 5% significance level, and an allocation ratio of 1, we estimated a sample size of n=102 infants.”*  
  The sample size calculation was performed using G*Power v3.1. The t-test distribution will be the appropriate distribution, whether doing the sample size calculation via a difference in means approach (in G*Power: t-tests family, “Means: Difference between two independent means (two groups)” option) or a linear regression approach (in G*Power: t-tests family, “Linear multiple regression: Fixed model, single regression coefficient” option). The details provided in the SAP allow the calculation of a Cohen’s D=0.6485, resulting in n=102. The equivalent effect size for the regression approach would be a partial coefficient of determination of the group allocation variable R ^2^=0.0957, resulting in a Cohen’s f ^2^=0.1052. We find the means and standard deviations to be more useful and interpretable to report than the partial R ^2^, but they are equivalent information that both result in n=102. This is true even if the number of predictors in the linear regression approach is increased from two (which would be equivalent to an unadjusted t-test) to seven (to include five prognostic variables). In the pilot analyses underlying the values going into the sample size calculations, the minimisation variables were not all available. However, we expect the inclusion of these minimisation variables to improve precision of the estimate, and so the sample size estimate based on the unadjusted approach is likely a more conservative number.  
- Baseline characteristics: How are (i) postmenstrual age at time of study (ii) cumulative pain exposure measured?
**Response: **We have updated the manuscript to include these details (see section ‘Baseline characteristics’).  
*“Postmenstrual age at time of study is measured as the sum of gestational age and postnatal age, both of which will be recorded in the neonate’s medical notes. Cumulative pain exposure is measured from each neonate’s clinical records as the total number of acute skin-breaking procedures, including heel lances, venepuncture, and intravenous cannulations, and aspirations (oropharyngeal or endotracheal) from time of birth to time of study.”*  
- Missing Data: Can you clarify whether you are planning to impute the outcome data? Or impute the covariates for the primary outcome model? It is not standard practice to impute outcome data.
**Response: **We intend to impute the covariates.  
- Additional analyses: The authors mention “intra- and inter-rater reliability” here. Could you clarify how PIPP-R scores from different raters will be used (i.e., averaged?) in the Outcome definitions section, as well as the number of raters? **Response: **We have updated the manuscript to include these details (see section ‘Outcome definitions’).  
*“Clinical pain scores will be calculated using the PIPP-R (Stevens et al., 2014). The score is composed of postmenstrual age, behavioural state, physiological changes (heart rate and oxygen saturation changes), as well as facial expression duration (brow bulge, eye squeeze and nasolabial furrow) in the 30 s post-procedure. The PIPP-R score is the sum of the ratings for each component of the assessment and has a range of 0–21. Videos and vital signs will be used to calculate the behavioural, heart rate and oxygen saturation components of the PIPP-R score; scores are not calculated live. The behavioural (facial expressions) components of the PIPP-R will be scored by researchers who have not been involved in the specific test occasion and who were blind to the procedure (sham heel lance or heel lance) and trial arm allocation. PIPP-R vital signs components will be calculated using automated scripts on blinded data. Each PIPP-R video will be initially scored by an individual rater. Subsequently, 20% of videos will be re-scored, either by a different researcher for the purpose of assessing inter-rater reliability or by the researcher who did the initial scoring to assess intra-individual reliability. The non-video components for the PIPP-R ratings will be calculated automatically and reliability will be assessed on the total PIPP-R score. If reliability is high (0.9 or greater), the initial video scoring will be used; if reliability is low, scores will be averaged.*”

# Reviewer report: Reviewer response for version 1

**DOI:** 10.21956/wellcomeopenres.21951.r71279

**Published:** 2024-01-31

**Authors:** Saminathan Anbalagan

**Affiliations:** 1 Lincoln Medical & Mental Health Center, New York, USA; 2 Pediatrics/Neonatology, University of South Alabama (Ringgold ID: 5557), Mobile, Alabama, USA

The Petal trial by Baxter et al. is a multicenter randomized controlled study investigating the effect of parental touch on acute neonatal pain and parental anxiety during heel lance procedure in newborns. It is an innovative study focusing on objective methods for identifying neonatal pain by evaluating EEG changes and physiological parameters. The trial employs a rigorous methodology, including a randomized parallel-group design with well-justified sample size calculations and statistical analytic methods. The Statistical Analysis Plan (SAP) meticulously outlines these, adhering to best practice guidelines to ensure reproducibility. 

 The authors are encouraged to consider the following minor feedbacks:

 - Please add gestational age in the abstract.

 - Please provide details on the standard of care (e.g., sucrose solution?) in each research site as it may affect the primary outcome and generalizability.

 - Authors must clarify the significant difference between the intervention and control groups as both have parental stroking at different time points.

 - Will the duration of heel lance be recorded? If multiple heel lances are required to obtain the blood, how will it be addressed?

 - Twins are known to have a differential response to pain. Will twins be included in this study? If yes, how will they be randomized?

 - The graphical representation of the timing of outcomes is well appreciated.  But please clarify if PIPP-R scoring will be a one-time measurement or multiple over a period of time. Consider obtaining the PIPP-R score at baseline (before parental touch) and also at the time of evaluating the primary outcome to increase the robustness of the EEG findings.

 - Multiple regression analysis is the appropriate statistical test for single time point comparisons. However, if repeated measures are obtained (PIPP-R), the authors must consider using a mixed-effects model or generalized estimating equations to analyze the effect of time on the outcome measured.

 - There are discrepancies between the ISRCTN details, ClinicalTrials.gov details and the manuscript here. Please clarify and unify the study design in these sites (e.g., HIE is not mentioned in the exclusion criteria in the trial registries but in this manuscript). Also, was the trial registered prior to patient enrollment? ISRCTN reports a 2020 start date, while registration in these registries happened in 2021?).

Is the work clearly and accurately presented and does it cite the current literature?

Yes

If applicable, is the statistical analysis and its interpretation appropriate?

Yes

Are all the source data underlying the results available to ensure full reproducibility?

Yes

Is the study design appropriate and is the work technically sound?

Yes

Are the conclusions drawn adequately supported by the results?

Yes

Are sufficient details of methods and analysis provided to allow replication by others?

Yes

Reviewer Expertise:

Neonatology, Neonatal Pain, Statistics.

I confirm that I have read this submission and believe that I have an appropriate level of expertise to confirm that it is of an acceptable scientific standard, however I have significant reservations, as outlined above.

# Response

**Published:** 2024-10-08

**Authors:** Luke Baxter

**Affiliations:** Department of Paediatrics, University of Oxford, Oxford, England, UK

The Petal trial by Baxter et al. is a multicenter randomized controlled study investigating the effect of parental touch on acute neonatal pain and parental anxiety during heel lance procedure in newborns. It is an innovative study focusing on objective methods for identifying neonatal pain by evaluating EEG changes and physiological parameters. The trial employs a rigorous methodology, including a randomized parallel-group design with well-justified sample size calculations and statistical analytic methods. The Statistical Analysis Plan (SAP) meticulously outlines these, adhering to best practice guidelines to ensure reproducibility.   The authors are encouraged to consider the following minor feedbacks:  

 - Please add gestational age in the abstract.  

**Response: **We have added gestational age to the abstract.  

*“The study involves in-patient neonates born at or after 35+0 weeks gestation with a postnatal age of ≤7 days, in two hospital research sites (John Radcliffe Hospital, Oxford, UK; Royal Devon and Exeter Hospital, Exeter, UK).”*  

 - Please provide details on the standard of care (e.g., sucrose solution?) in each research site as it may affect the primary outcome and generalizability.  

**Response: **We have added text to detail standard of care in each research site (see section ‘Trial design and framework’).  

*“In both sites, standard of care involves offering all neonates comfort measures including swaddling and non-nutritive sucking, in line with local practice guidelines.”*  

 - Authors must clarify the significant difference between the intervention and control groups as both have parental stroking at different time points.  

**Response: **We have reworded the description of the two groups to clarify how they are distinct (see section ‘Trial design and framework’).

*“This is a superiority trial comparing standard of care and parental stroking performed before a heel lance (the intervention group) to standard of care alone before a heel lance (the control group). Note, in the control group, the parents also stroked their baby after the heel lance and after the baby’s responses to the heel lance were recorded; thus, the post-procedural parental stroking in the control group had no impact on the heel lance procedure or the recorded responses to the heel lance.”*  

 - Will the duration of heel lance be recorded? If multiple heel lances are required to obtain the blood, how will it be addressed?  

**Response: **The duration of the heel lance event is almost instantaneous, the blade is spring loaded and once triggered, it makes the incision within a fraction of a second. It is highly standardised medical device, so there is no meaningful variability in heel lance durations. We have updated the text to better describe the heel lance procedure (see section ‘Trial design and framework’).  

*“The heel lance procedure involves using a small, spring-loaded lancet with a blade that, within milliseconds, creates a small cut that allows blood to be collected for testing.”*  

 If multiple heel lances are required, only the first one will be used. The pain responses to subsequent lances will likely be influenced by the initial heel lance. We have clarified this in the text (see section ‘Outcome definitions’).  

*“If more than one heel lance is required during the blood test procedure, only the first heel lance will be used for calculating the three neonate’s outcomes: EEG, PIPP-R, tachycardia. Any subsequent heel lances will not occur within the 30 s after the first heel lance, as these 30 s are part of the response estimation.”*  

 - Twins are known to have a differential response to pain. Will twins be included in this study? If yes, how will they be randomized?  

**Response: **We won’t exclude twins from this study. If twins are recruited into the trial, we will randomise them independently.  

 - The graphical representation of the timing of outcomes is well appreciated. But please clarify if PIPP-R scoring will be a one-time measurement or multiple over a period of time. Consider obtaining the PIPP-R score at baseline (before parental touch) and also at the time of evaluating the primary outcome to increase the robustness of the EEG findings.  

**Response: **PIPP-R scoring will be a one-time measurement. The calculation of PIPP-R intrinsically involves a measurement of post-procedure response relative to a pre-procedure baseline.  

 - Multiple regression analysis is the appropriate statistical test for single time point comparisons. However, if repeated measures are obtained (PIPP-R), the authors must consider using a mixed-effects model or generalized estimating equations to analyze the effect of time on the outcome measured.  

**Response: **The PIPP-R measure is a single time point outcome measure.  

 - There are discrepancies between the ISRCTN details, ClinicalTrials.gov details and the manuscript here. Please clarify and unify the study design in these sites (e.g., HIE is not mentioned in the exclusion criteria in the trial registries but in this manuscript). Also, was the trial registered prior to patient enrollment? ISRCTN reports a 2020 start date, while registration in these registries happened in 2021?).  

**Response: **Thank you for highlighting these discrepancies between the manuscript and the trial registrations. To clarify, the information in this SAP manuscript is consistent with the detailed protocol and SAP documents that were attached to the ISRCTN registration. However, the information on the trial registration webpages had not been updated to be in line with the final protocol and SAP attachments. We have now requested updates to the ISRCTN registration webpage to be consistent with the other documents and these should be implemented imminently.  

 Please note that the dates in the plain English summary are based on the overall study start and end dates rather than the recruitment dates. The ISRCTN webpage in error currently states the original planned recruitment dates of 2020-2023 - however due to the covid pandemic the trial was delayed in its development, submission for regulatory approvals, and recruitment. Recruitment therefore started in September 2021 and continued until February 2023 - we can confirm that the trial was registered before participant enrolment. We have requested this information be updated in the ISRCTN registration page. Similarly, the updates are in progress to the Clinicaltrials.gov record.

# Reviewer report: Reviewer response for version 1

**DOI:** 10.21956/wellcomeopenres.21951.r71287

**Published:** 2024-01-04

**Authors:** Abhishek Sharma

**Affiliations:** 1 Department of Physiotherapy, Arogyam Institute of Paramedical and Allied Sciences (Affiliated to H.N.B. Uttarakhand Medical Education University), Roorkee, Uttarakhand, India

Abstract: The current abstract does not adhere to the prescribed guidelines of the journal, lacking essential information about proposed results. Notably, it omits crucial details in the methods section, including participants' age, randomization specifics, study type, setting, and location. Furthermore, authors are encouraged to incorporate MeSH terms for keywords using the MeSH Browser.

 Introduction: The introduction lacks explicit hypothesis testing.

 Methods: Discrepancies exist between the ClinicalTrials.gov registration and manuscript regarding the study design, necessitating clarification. The manuscript is deficient in procedure details such as duration, repetition per session, and the specific day of neonatal performance.

 Analysis of the treatment groups reveals a lack of substantial differences, as both groups seemingly receive identical treatments regardless of the order (Group 1 pre-procedure touch, Group 2 post-procedure touch). Authors are advised to introduce distinguishing elements to enhance the protocol's robustness.

 Incorporating the SPIRIT flow diagram and SPIRIT Checklist in the Methods section and manuscript annexures, respectively, is recommended. Authors must ensure adherence to the SPIRIT Checklist points in the protocol manuscript.

 The current CONSORT Diagrams utilized require modification. Authors are urged to adopt the non-pharmacological extension of CONSORT in its latest version for this study.

 The manuscript reveals a lack of interim analysis and associated timings, raising concerns about potential publication bias and researcher bias.

 Furthermore, while the PIPP-R version is employed as an outcome measure, the inclusion criteria for premature neonates are not explicitly stated, leaving a gap in information regarding gestational age. Authors are strongly encouraged to address this issue by specifying the gestational age criteria or considering alternative outcome measures such as NIPS or NPASS.

 The utilization of the State-Trait Anxiety Inventory (STAI) for anxiety measurement in neonates requires justification. The authors are advised to provide a rationale for choosing this specific tool in the context of neonatal anxiety.

 Additionally, a significant portion of the references cited by the authors is notably outdated. It is recommended that the authors update their references to include more recent sources, preferably from the last 5-7 years, to ensure the incorporation of current and relevant literature.

Is the work clearly and accurately presented and does it cite the current literature?

Partly

If applicable, is the statistical analysis and its interpretation appropriate?

Partly

Are all the source data underlying the results available to ensure full reproducibility?

Partly

Is the study design appropriate and is the work technically sound?

Yes

Are the conclusions drawn adequately supported by the results?

Yes

Are sufficient details of methods and analysis provided to allow replication by others?

Yes

Reviewer Expertise:

Pediatrics, Physiotherapy, Research methodology, Statistics

I confirm that I have read this submission and believe that I have an appropriate level of expertise to confirm that it is of an acceptable scientific standard, however I have significant reservations, as outlined above.

# Response

**Published:** 2024-10-08

**Authors:** Luke Baxter

**Affiliations:** Department of Paediatrics, University of Oxford, Oxford, England, UK

Abstract: The current abstract does not adhere to the prescribed guidelines of the journal, lacking essential information about proposed results. Notably, it omits crucial details in the methods section, including participants' age, randomization specifics, study type, setting, and location. Furthermore, authors are encouraged to incorporate MeSH terms for keywords using the MeSH Browser.  

**Response: **Thank you for highlighting these omissions. We have updated the abstract to include these details. To keep within Journal word limitations (max 300 words), we have removed detail from the background and conclusions sections of the abstract.  

*“ **Background**. Infants undergo multiple clinically required painful procedures during their time in hospital, and there is an increasing desire from both parents and clinical staff to have parents directly involved in their newborn’s pain relief. To avoid biases due to selective analysis and reporting, a clinical trial’s statistical analysis plan (SAP) should be finalised and registered prior to dataset lock and unblinding. Here, we outline the SAP for the Petal trial, which was registered on the ISRCTN registry prior to dataset lock and unblinding.*  

***Methods*** *. The Petal trial is a multicentre, individually randomised, parallel-group interventional superiority trial. The study involves in-patient neonates born at or after 35+0 weeks gestation with a postnatal age of ≤7 days, in two hospital research sites (John Radcliffe Hospital, Oxford, UK; Royal Devon and Exeter Hospital, Exeter, UK). The primary objective is to investigate the potential efficacy of a non-pharmacological parent-led stroking intervention on reducing the magnitude of neonates’ noxious stimulus-evoked brain activity. The primary outcome is the neonate’s brain activity recorded using electroencephalography (EEG) in response to a heel lance blood sampling procedure. Secondary outcomes include neonatal clinical pain scores and tachycardia, and parental anxiety. The study hypothesis is neonates’ pain responses and parents’ anxiety scores are lower in the intervention group. Randomisation will be via a minimisation algorithm to maintain balance in five prognostic factors.*  

***Conclusion*** *. Paediatric pain trials have been highlighted by regulatory bodies as an important and challenging topic, with interest increasing in brain imaging outcomes. The Petal trial, to which this SAP relates, is part of a larger effort of establishing a brain-based EEG outcome measure of infant pain for use in clinical trials. This SAP is thus likely to be of interest to those in academia, pharmaceutical companies, and regulatory bodies.*  

***Trial registration*** *. ClinicalTrials.gov Identifier: NCT04901611; ISRCTN14135962.”*  

 We have also requested for the journal to update our keywords to the following (MeSH terms are highlighted in **bold**):  

***Pain*** *, **Infant Care**, **Therapeutic Touch**, **Parents**, PIPP-R, **Electroencephalography**, **Biomarkers**, **Randomized Controlled Trial***  

 Introduction: The introduction lacks explicit hypothesis testing.  

**Response: **We have added the hypothesis to the section ‘Objectives’.  

*“For all primary and secondary outcomes, the null (* *H* *0* *) and alternative (* *H* *1* *) hypotheses are as follows.* *H* *0* *:* *μ* *1* *=* *μ* *2* *or* *OR* *1* *=* *OR* *2* *;* *H* *1* *:* *μ* *1* *≠* *μ* *2* *or* *OR* *1* *≠* *OR* *2* *, where* *μ* *1* *and* *μ* *2* *are the means (for EEG, PIPP-R, and STAI-S) and* *OR* *1* *and* *OR* *2* *are the odds ratios (for tachycardia) of the intervention and control groups.”.*  

 Methods: Discrepancies exist between the ClinicalTrials.gov registration and manuscript regarding the study design, necessitating clarification. The manuscript is deficient in procedure details such as duration, repetition per session, and the specific day of neonatal performance.  

**Response: **Thank you for highlighting these discrepancies between the manuscript and the trial registration. To clarify, the information in this SAP manuscript is consistent with the detailed protocol and SAP documents that were attached to the ISRCTN registration. However, the information on the webpage itself had not been updated to be in line with the final protocol and SAP attachments. We have now requested updates to the registration webpage to be consistent with the other documents, and these should be implemented imminently.   The duration of the heel lance event is almost instantaneous, the blade is spring loaded and once triggered, it makes the incision within a fraction of a second. It is highly standardised medical device, so there is no meaningful variability in heel lance durations. We have added the following to better explain the heel lance procedure (see section ‘Trial design and framework’):  

*“The heel lance procedure involves using a small, spring-loaded lancet with a blade that, within milliseconds, creates a small cut that allows blood to be collected for testing.”*  

 If multiple heel lances are required, only the first one will be used. The pain responses to subsequent lances will likely be influenced by the initial heel lance. We have clarified this in the text (see section ‘Outcome definitions’).  

*“If more than one heel lance is required during the blood test procedure, only the first heel lance will be used for calculating the three neonate’s outcomes: EEG, PIPP-R, tachycardia. Any subsequent heel lances will not occur within the 30 s after the first heel lance, as these 30 s are part of the response estimation.”*  

 There is no specific day for assessments. Neonates are studied when they clinically require heel lance procedures. No neonates underwent heel lances for the purposes of research, and the undertaking of research was designed to have minimal impact on standard clinical processes.  

 Analysis of the treatment groups reveals a lack of substantial differences, as both groups seemingly receive identical treatments regardless of the order (Group 1 pre-procedure touch, Group 2 post-procedure touch). Authors are advised to introduce distinguishing elements to enhance the protocol's robustness.  

**Response: **We have reworded the description of the two groups to clarify how they are distinct (see section ‘Trial design and framework’).  

*“This is a superiority trial comparing standard of care and parental stroking performed before a heel lance (the intervention group) to standard of care alone before a heel lance (the control group). Note, in the control group, the parents also stroked their baby after the heel lance and after the baby’s responses to the heel lance were recorded; thus, the post-procedural parental stroking in the control group had no impact on the heel lance procedure or the recorded responses to the heel lance.”*  

 Incorporating the SPIRIT flow diagram and SPIRIT Checklist in the Methods section and manuscript annexures, respectively, is recommended. Authors must ensure adherence to the SPIRIT Checklist points in the protocol manuscript.  

**Response: **The SPIRIT checklist and flow diagram is specifically for a clinical trial protocol. Given this is a statistical analysis plan, the relevant guideline and checklist is the 2017 JAMA guidance document and checklist for statistical analysis plans in clinical trials. We have incorporated these guidelines throughout the manuscript.  

 The current CONSORT Diagrams utilized require modification. Authors are urged to adopt the non-pharmacological extension of CONSORT in its latest version for this study.  

**Response: **Thank you for bringing our attention to this CONSORT extension. We will be sure to follow these CONSORT extension guidelines, checklist, and flow diagram when reporting our clinical trial.  

 The manuscript reveals a lack of interim analysis and associated timings, raising concerns about potential publication bias and researcher bias.  

**Response: **We address these risks of bias by having pre-registered the trial and publishing a protocol. These additional registrations and publications are designed specifically to publicly detail the existence and specifics of the trial, which directly addresses these types of biases.

 Furthermore, while the PIPP-R version is employed as an outcome measure, the inclusion criteria for premature neonates are not explicitly stated, leaving a gap in information regarding gestational age. Authors are strongly encouraged to address this issue by specifying the gestational age criteria or considering alternative outcome measures such as NIPS or NPASS. 

**Response: **PIPP-R has been validated for use in the age range of neonates included in the Petal trial. Despite the name of Premature Infant Pain Profile, the tool has been validated for use in both term and pre-term neonates. Worth noting is that one of the contextual inputs of the PIPP-R is the PMA at study, which includes the following categories:

 -  >= 36 weeks

 -  32 weeks to 35+6 weeks

 -  28 weeks to 31+6 weeks

 -  < 28 weeks

 The neonates in the Petal trial all have ages that fall in the first two categories.  

 The utilization of the State-Trait Anxiety Inventory (STAI) for anxiety measurement in neonates requires justification. The authors are advised to provide a rationale for choosing this specific tool in the context of neonatal anxiety.  

**Response: **The STAI is used for measuring parental anxiety. The STAI is well established and well validated for measuring anxiety in adults in the literature. We have clarified this in the manuscript (see section ‘Outcome definitions’).  

*“The State-Trait Anxiety Inventory (STAI) is a widely used, validated and reliable assessment for anxiety (Spielberger et al., 1983). The STAI-S scale (anxiety state) consists of 20 statements. It will require the parents to rate their anxiety at a particular moment in time (pre- and post-procedure for this trial) and is used to determine the parental level of anxiety induced by stressful procedures. Each question is rated on a 4-point Likert scale. STAI-S thus has a range of 20–80.”*  

 Additionally, a significant portion of the references cited by the authors is notably outdated. It is recommended that the authors update their references to include more recent sources, preferably from the last 5-7 years, to ensure the incorporation of current and relevant literature.  

**Response: **As of the present date, publications from more than seven years ago are those published prior to 2017. This includes 9 of 19 references in our version 1 submission. We believe it is appropriate to retain all these older papers. There are no reasonable or necessary substitutions. Two papers are the original papers establishing our outcome measures for PIPP-R (published in 2014) and STAI (published in 1983), and there cannot be a more recent paper establishing these tools as landmark papers establishing a tool are by their nature one of a kind. Two papers are guidelines and recommendations papers, including IMMPACT recommendations (published in 2008) and CONSORT statement (published in 2010), for which there is no appropriate replacement. One reference is an FDA event (in 2016) for which there is no reasonable substitute. One paper referenced from 1997 was one of two papers required to justify sample size planning; there are no additional papers; we already included the second paper from 2018 (thus, we include a paper from 2016 and 2018 to justify the sample size); and removing the older publication would not be appropriate. Two papers are classic well-known and well-respected meta-analyses from 2010 and 2014 for which there is no better publication to substitute. The only reference we think we could consider substituting is the Baker 2016 paper, but this would not improve the quality of the manuscript; this Baker publication is a high-quality publication in Nature of over 1500 researchers surveyed, it is a reference in the introduction, and replacing it simply because it is from 2016 would not be improve this manuscript.
